# Improved segmentation of deep brain grey matter structures using magnetization transfer (MT) parameter maps

**DOI:** 10.1016/j.neuroimage.2009.03.053

**Published:** 2009-08-01

**Authors:** Gunther Helms, Bogdan Draganski, Richard Frackowiak, John Ashburner, Nikolaus Weiskopf

**Affiliations:** a MR-Research in Neurology and Psychiatry, University Medical Center, Göttingen, Germany; b Wellcome Trust Centre for Neuroimaging, UCL Institute of Neurology, University College London, 12 Queen Square, London, WC1N 3BG, UK; c Max Planck Institute for Human Cognitive and Brain Sciences, Leipzig, Germany; d Département d'Études Cognitives, Ecole Normale Supérieure, Paris, France; e Laboratory of Neuroimaging, Fondazione Santa Lucia IRCCS, Roma, Italy

## Abstract

Basal ganglia and brain stem nuclei are involved in the pathophysiology of various neurological and neuropsychiatric disorders. Currently available structural T1-weighted (T1w) magnetic resonance images do not provide sufficient contrast for reliable automated segmentation of various subcortical grey matter structures. We use a novel, semi-quantitative magnetization transfer (MT) imaging protocol that overcomes limitations in T1w images, which are mainly due to their sensitivity to the high iron content in subcortical grey matter. We demonstrate improved automated segmentation of putamen, pallidum, pulvinar and substantia nigra using MT images. A comparison with segmentation of high-quality T1w images was performed in 49 healthy subjects. Our results show that MT maps are highly suitable for automated segmentation, and so for multi-subject morphometric studies with a focus on subcortical structures.

## Introduction

In the field of computational neuroanatomy, a substantial amount of work has been devoted to automated tissue classification and segmentation in the analysis of cerebral cortex with magnetic resonance imaging (Ashburner et al., 2003; May and Gaser, 2006). However, the accurate segmentation of subcortical structures such as the basal ganglia (putamen, caudate, pallidum, subthalamic nucleus (STN), substantia nigra pars compacta (SNc)) and thalamus for in-vivo studies of normal development and disease is still challenging (Wonderlick et al., 2009). Various neurological and neuropsychiatric disorders are associated with basal ganglia and/or thalamic dysfunction (McGuire et al., 1994; Meunier et al., 2003; Utter and Basso, 2008). The basal ganglia receive projections from almost the entire cortex and channel output via thalamic relays (for a review see Graybiel, 1995; Haber, 2003; Middleton and Strick, 2002; Parent and Hazrati, 1995; Parent et al., 2000; Percheron et al., 1994), so that the basal ganglia and thalamus have a major influence on sensori-motor, limbic and cognitive information processing (Hoover and Strick, 1999; Middleton and Strick, 1994, 1997, 2000, 2002). Detailed anatomical knowledge of subcortical structures is essential for early diagnosis of neurological and neuropsychiatric disorders, such as typical vs. atypical forms of Parkinson's syndrome, Huntington's disease, dystonia, various tremor forms, Tourette's syndrome and schizophrenia (for detailed review see Utter and Basso, 2008).

Currently available surface, shape or volume based methods for analysis of anatomical magnetic resonance images (MRI) are commonly applied to T1-weighted (T1w) three-dimensional (3D) data sets. The somewhat discouraging segmentation of subcortical structures may be largely explained by their specific anatomical properties and the associated limitations of standard MRI methods. Unlike the layered structure of the cortex, basal ganglia and thalamic neuronal nuclei are connected by complex and intertwined axonal tracts below the resolution limits of standard T1w imaging (∼ 1 mm), thus reducing contrast by partial volume averaging. In addition, a high iron content of the major midbrain nuclei (SNc, red and subthalamic nuclei, pallidum and putamen, for a review see Haacke et al., 2005) further shortens T1. These structures exhibit reduced contrast from white matter (WM) in T1w images and are thus often misclassified by segmentation algorithms.

There is an emerging need for high-resolution MR images that provide sufficient contrast for reliable automated segmentation of subcortical structures. To this end, we used parameter maps based on magnetization transfer (MT) contrast. Motivated by preliminary single subject results (Helms et al., 2008b), this study tests the hypothesis that MT maps provide a more reliable segmentation of subcortical structures than standard T1w images. We compare the segmentation results derived from MT maps with those from an established T1w imaging method (3D Modified Driven Equilibrium Fourier Transform, MDEFT), in a cohort of 49 subjects. Considerable improvements were seen in putamen, pallidum, SNc, and pulvinar.

## Methods

49 healthy adults (age 18–85; male:female = 24:25) were examined on a 3T whole-body MRI system (Magnetom TIM Trio, Siemens Medical Systems, Erlangen, Germany). Informed written consent was obtained as supervised by the local Ethics committee.

### MR imaging

All 3D datasets were acquired in sagittal orientation with 1 mm isotropic resolution (176 partitions, field of view (FOV) = 256 × 240 mm^2^, matrix 256 × 240 × 176) and non-selective excitation. T1w structural imaging was performed using a 3D MDEFT sequence (TR = 7.92 ms, TE = 2.48 ms, TI = 910 ms [symmetrically distributed around the inversion pulse; quot = 50%], flip angle *α* = 16°, fat saturation, bandwidth 195 Hz/Pixel, acquisition time approx. 13 min; Deichmann et al., 2004) with compensation for B1 inhomogeneities.

MT maps were calculated from a multi-parameter protocol based on a 3D multi-echo fast low angle shot (FLASH) sequence (Weiskopf and Helms, 2008). Three co-localised 3D multi-echo FLASH datasets were acquired with predominantly proton density weighting (PDw: TR/*α* = 23.7 ms/6°), T1w (18.7 ms/20°), and MTw (23.7 ms/6°; excitation preceded by an off-resonance Gaussian MT pulse of 4 ms duration, 220° nominal flip angle, 2 kHz frequency offset) in a total acquisition time of approx. 19 min. The signals of six equidistant bipolar gradient echoes (at 2.2 ms to 14.7 ms echo time) were averaged to increase the SNR (Helms and Dechent, 2009). A rather high acquisition bandwidth of 425 Hz/pixel was chosen to keep the susceptibility-related geometric distortions in brain and the chemical shift displacement of fat signals below one pixel. To speed up the acquisition, GRAPPA parallel imaging with an acceleration factor of two in the phase-encoding direction (anterior–posterior) and 6/8 partial Fourier in the partition direction (left–right) were employed. Semi-quantitative MT parameter maps, corresponding to the additional saturation created by a single MT pulse, were calculated by means of the signal amplitudes and T1 maps (Helms et al., 2008a), eliminating the influence of relaxation and B1 inhomogeneity (Helms et al., 2008b).

### Data processing

Data processing and analysis were performed with the freely available Statistical Parametric Mapping software (SPM5; Wellcome Trust Centre for Neuroimaging, London, UK; http://www.fil.ion.ucl.ac.uk/spm) running under Matlab 7 (Mathworks, Sherborn, MA, USA). The pre-processing involved segmentation and spatial normalisation of the three-dimensional image data followed by scaling with the Jacobian determinants (Ashburner and Friston, 2000).

The structural data were segmented into different tissue classes — grey matter (GM), white matter (WM) and non-brain voxels (CSF, skull), using the unified segmentation approach in SPM5 (Ashburner and Friston, 2005). To obtain greater anatomical precision of inter-subject alignment, spatial normalisation was performed using the Dartel algorithm (Ashburner, 2007), with default settings. This procedure iteratively aligns the grey and white matter images with their common respective averages, which become increasingly crisp as registration proceeds (Ashburner and Friston, 2009). Subsequently, the tissue maps were scaled by the Jacobian determinants from the final normalisation step. When images are warped, some regions will be expanded and others contracted. Scaling the warped images by the Jacobian determinants preserves the total volume of tissue in each structure (Ashburner and Friston, 2000).

We compared the population averages of grey matter probability maps generated from the MDEFT and MT maps, focussing on subcortical deep brain structures. For statistical comparison, the GM segments were smoothed by convolution with an isotropic Gaussian kernel of 3 mm full-width at half maximum (FWHM). We used a paired *t*-test with search volume reduced to the whole extent of basal ganglia, thalamus and brainstem, i.e., the subcortical region of interest. Anatomical topography and spatial extent were based on atlases provided by FreeSurfer (Desikan et al., 2006). Regional differences between MT and MDEFT GM segments were examined by creating voxel-wise statistical parametric maps (SPMs) for the whole extent of the predefined search volume using the General Linear Model and Random Fields Theory. Significance levels were set at *p* < 0.05 after family-wise error (FWE) correction for multiple comparisons.

In addition to comparing the GM segmentation results, the contrast- and signal-to-noise ratio (CNR, SNR) in the original MDEFT images and MT maps were compared in 10 randomly chosen subjects by a manual region-of-interest (ROI) analysis. Using the MRIcron tool (www.sph.sc.edu/comd/rorden/mricro.html), spherical ROIs (of 4 mm maximum radius) were placed on the individual MDEFT images bilaterally in the anterior pallidum, the caudate head, and the genu of the corpus callosum (as typical WM). Neighbouring structures were chosen to reduce the potential bias imposed by B1 inhomogeneities. The standard deviations (SD) across the ROIs were chosen as a conservative estimate for the noise notwithstanding underlying structural heterogeneity. The SNR was defined as the ratio of the mean signal over the SD in the ROI. The CNR between two ROIs was defined as the difference in the means over the square root of the mean variance in the ROIs. CNR and SNR of MDEFT images and MT maps were compared by a paired two-sample *t*-test (*p* < 0.05).

## Results

**Fig. 1:**
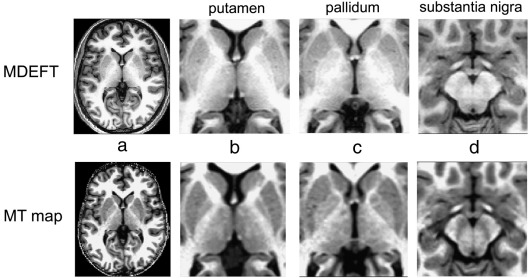
Example of single subject T1w MDEFT (upper row) and MT map (bottom row) data in Montreal Neurological Institute (MNI) standard space. (a) transverse view of whole head; (b) putamen, (c) pallidum, (d) substantia nigra shown as a zoomed view. Consistent windowing was based on whole brain histograms (MDEFT: [35 a.u.–470 a.u.]; MT [0 p.u.–1.9 p.u.]).

Segmented MT maps showed clearly improved delineation of substantia nigra pars compacta (SNc), caudal (posterior) putamen, and rostral (anterior) pallidum, as well as the lateral pulvinar nuclei in the thalamus. These regions exhibited little contrast to white matter on the T1w MDEFT images, whereas they appeared more hypointense on the MT maps, resulting in a higher contrast (for an example, see Fig. 1). The contrast also improved in the periaqueductal grey, and the dentate nuclei of the cerebellum (not shown). In addition, the MT maps showed a better delineation of WM laminae embedded in GM structures, as seen in the thalamus (Fig. 1b).

CNR values obtained by ROI analysis corroborated the higher visual MT contrast between pallidum and WM with a CNR = 6.2 ± 1.2 (mean ± SD) for MT vs. 3.9 ± 1.5 for MDEFT (*p* < 0.0015). The same CNR was found for both methods between caudate and WM (9.2 ± 0.8 for MT; 9.3 ± 2.2 for MDEFT, *p* = 0.93). The SNR of MDEFT, however, was consistently higher (pallidum: 13.8 ± 3.6; caudate: 12.0 ± 2.0; genu: 23.2 ± 4.2) than that of MT maps (8.3 ± 3.6; 10.4 ± 2.7; 12.7 ± 1.0) with all *p* < 0.013. The difference in SNR can be attributed to the longer acquisition time of the MDEFT image (∼ 13 min) compared to the MT map (∼ 6 min per data set).

**Fig. 2:**
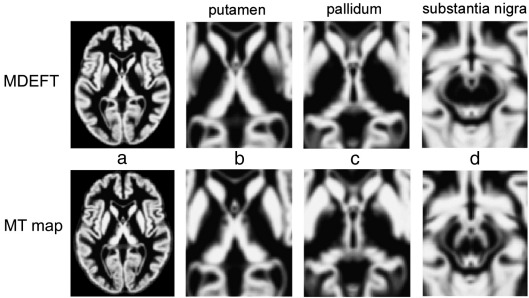
Population average maps (*n* = 49) of gray matter probability in MNI standard space corresponding to the single subject images in Fig. 1: (a) full transverse view, (b) putamen, (c) pallidum, (d) substantia nigra. MT map (bottom row) showing improved segmentation of the basal ganglia compared to T1w MDEFT (upper row).

**Fig. 3:**
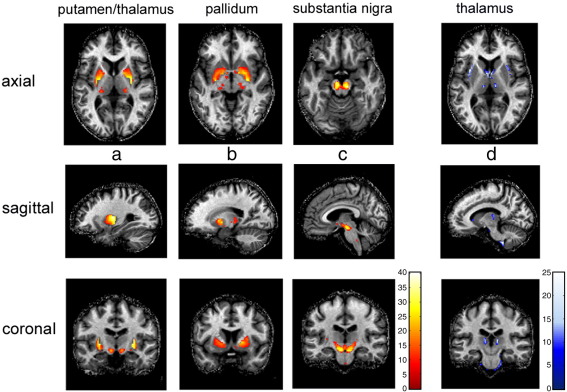
Statistical comparison between MDEFT- and MT-based population averaged GM probability maps in MNI standard space. *t*-test was performed on a subcortical ROI and voxels exceeding a threshold of *p* < 0.05 (FWE corrected) are displayed on a spatially normalised MT map of an individual. Columns a–c show increases in GM probability based on MT maps; column d decreases. The subregions of the thalamus were assigned to the lateral pulvinar (a,b top) and parts of the internal medullary lamina (d).

Consistent with the visual inspection of MT maps and CNR comparisons, the population averaged GM maps generated from the MT maps revealed a higher probability of identifying iron-rich midbrain nuclei than the ones based on MDEFT images, i.e., posterior putamen (Fig. 2b), anterior pallidum (Fig. 2c), and SNc, (Fig. 2d). In addition, a larger portion of the pulvinar nuclei of the thalamus could be assigned to GM (Fig. 2b). The statistical comparison in the subcortical ROI corroborated the results, indicating significantly increased GM probabilities for the MT-based maps in the putamen, pallidum, thalamus, and SNc (*p* < 0.05 FWE corrected). For details see Table 1 and Figs. 3a–c. Little or no improvement was seen in posterior pallidum, the subthalamic and red nuclei, and the ventro-anterior and ventro-lateral nuclei of the thalamus (nomenclature according to Hirai and Jones, 1989). These GM structures contain a larger number of axons, which reduce the contrast on MDEFT and MT maps alike. Between the medio-dorsal, ventro-lateral and pulvinar nuclei in the thalamus, the MT-based segmentation showed a lower GM probability than the MDEFT-based segmentation (*p* < 0.05 FWE corrected Fig. 3d). The significant reductions in sub-insular WM and caudate veins were consistent with anatomy.

## Discussion

We demonstrate robust and reliable delineation of putamen, pallidum, SNc, as well as pulvinar and medio-dorsal thalamus using automated segmentation of MT maps (Figs. 2 and 3). The novelty of our study builds on the generation of high-resolution semi-quantitative MT maps with improved contrast between tissue classes in subcortical regions and their use for automated tissue segmentation. Our results show the suitability of MT maps for automated segmentation, allowing for multi-subject morphometric studies.

Segmentation methods rely on the contrast between grey and white matter, which is primarily based on the presence of myelinated axons. MT is considered a more direct measure of the content of myelin (and other macromolecules) than T1 relaxation, which mainly reflects the physical properties of tissue water. In addition, the presence of iron in GM reduces the T1 relaxation time, and thus the contrast to WM in standard T1w imaging. Note that MT contrast has been commonly reported as “MT ratio” (Dousset et al., 1992), a parameter that shows a residual T1 dependence. Here, we calculated a novel semi-quantitative parameter, the MT saturation, which separates MT from T1 effects (Helms et al., 2008b). Such MT maps are corrected for confounding influences of proton density and T1 relaxation changes. As an additional benefit for automated segmentation, the MT maps are automatically corrected for inhomogeneities in the transmit and receive radio-frequency (B1) fields (Helms et al., 2008b). These cause a well-known spatial bias that impairs GM/WM segmentation in T1w images unless it is corrected at the processing or acquisition stage (Vovk et al., 2007). This is of particular importance at high field strengths and when using surface coils. Iron accumulation in tissue, during normal ageing (Ogg and Steen, 1998) or associated with disease (Berg and Youdim, 2006; Schenk et al., 2006), shortens T1 relaxation time and systematically interferes with automated segmentation by degrading the T1 contrast. By excluding the above mentioned confounds, which affect T1w data, we expect the interpretation of MT-based morphometric results to be more straightforward. We thus ascribe the significantly reduced GM probability in central thalamus to partial volume contributions of the internal medullary lamina (Hirai and Jones, 1989).

MT maps are potentially suitable for highly accurate anatomical localisation of subcortical structures in individuals, e.g., for target definition in individuals undergoing deep brain stimulation (DBS). The main advantages are an excellent GM/WM contrast in the ponto-mesencephalic area, short acquisition time (∼ 19 min) and high isotropic resolution of 1 mm. Further, we demonstrate major improvement in segmentation results for a cohort of 49 subjects using state-of-the-art, freely available software tools. This allows a reliable automated classification of tissue in subcortical structures involved in the pathogenesis of a wide range of neurological and neuropsychiatric disorders (typical vs. atypical forms of Parkinson's syndrome, Huntington's disease, dystonia, various tremor forms, Tourette's syndrome and schizophrenia, for detailed reviews see McGuire et al., 1994; Meunier et al., 2003; Utter and Basso, 2008). Reproducible morphometric characterisation of whole brain grey matter tissue combined with accurate automated assessment of deep brain nuclei will contribute to understanding the pathophysiology, establishing non-invasive biomarkers for early diagnosis and therapy monitoring.

## Conclusion

We demonstrate improved segmentation results for putamen, pallidum, SNc and thalamus using automated state-of-the-art segmentation of MT maps compared to optimised T1w scans. This method is of benefit for accurate anatomical localisation in individuals and for whole brain morphometric studies focussing on subcortical structures with high iron content.

## Figures and Tables

**Table 1:** 

Analysis	Region	Left hemisphere coordinates (mm)			*t*-score	Right hemisphere coordinates (mm)			*t*-score
*x*	*y*	*z*	*x*	*y*	*z*
MT > MDEFT	Putamen	− 27	− 14	5	28.11	28	− 10	7	40.2
SNc	− 6	− 18	− 14	31	9	− 22	− 14	35.2
GP	− 12	2	0	25.5	20	0	− 2	27.6
Thalamus	− 21	− 27	3	14.3	21	− 25	5	15
MT < MDEFT	Thalamus	− 9	− 24	6	14.6	10	− 22	8	18.5

Comparison between MDEFT- and MT-based population averaged GM maps in subcortical ROI: peak voxels. Coordinates are given in MNI standard space. SNc = substantia nigra pars compacta; GP = globus pallidus.
